# Posterior segment ocular manifestations of HIV/AIDS patients

**Published:** 2014-09-25

**Authors:** C Chiotan, L Radu, R Serban, C Cornăcel, M Cioboată, A Anghelie

**Affiliations:** * "Prof. Dr. Matei Bals" National Institute of Infectious Diseases, Bucharest, Department of HIV / AIDS, Office of Ophthalmology; ** Ophthalmology Emergency Hospital, Bucharest

**Keywords:** posterior segment ocular manifestations, microangiopathy HIV, CMV retinitis

## Abstract

Abstract

Human immunodeficiency virus (HIV) has the ability to affect any organ in the body. In 70% of HIV -infected patients ocular manifestations were observed, these, in the vast majority reflect the systemic disease and may be the first signs of disseminated infections.

Aim: The purpose of this paper is to determine the prevalence of posterior segment ocular manifestations in HIV / AIDS (Acquired Immunodeficiency Syndrome) patients.

Method: The study is retrospective, conducted in the Cabinet of Ophthalmology of "Matei Bals" Infectious Diseases Hospital in Bucharest, during the period 1 August 2007 - 1 August 2013 . Each patient was examined thoroughly at the biomicroscope ocular slit by using 90D microscope lens and 20D indirect lens after the administration of topical mydriatics.

Results: 348 patients with HIV/AIDS and ocular disorders were followed. There was a high number of children and young people with HIV who had eye disorders (194 patients aged between 14 and 25 years). 44.25% of patients had posterior segment ocular damage, 17.55% of them had the anterior segment affected. 22.90% of the 131 patients with compromised posterior segment microangiopathy have been diagnosed with HIV / AIDS.

Conclusions: Doctors should be aware of the existence of ocular damage in HIV / AIDS and to emphasize the importance of regular ophthalmologic examination of patients with HIV / AIDS.

Abbreviations: HIV- human immunodeficiency virus, AIDS- acquired immunodeficiency syndrome, CMV retinitis-cytomegalovirus retinitis

## Introduction

HIV / AIDS is undoubtedly a multisystemic disease, but eye disease occurs in up to 70% of cases during the natural history of infection. Ophthalmic manifestations of HIV-associated spectrum are very broad and extend from a simple blepharitis to blindness induced by CMV retinitis (cytomegalovirus retinitis) [**1**].

 On June 30, 2012 there were a number of 11 189 patients of HIV / AIDS alive in Romania. This means that approximately 7,800 patients may have had the ocular manifestations of the disease [**2**].

 Ophthalmic pathology in those infected with HIV is due to opportunistic infections, vascular anomalies, neoplasms, diseases induced by specific medication or neuroophtalmic damage. Among these, opportunistic infections are the main cause of morbidity and ocular disease with the highest potential of destruction in patients with AIDS [**4**].

 Coinfection with CMV occurs in 75-85% of the patients with HIV infection, of which more than half develop CMV retinitis. Despite the high incidence, there are difficulties regarding the therapeutic approach and the results can only be satisfactory even with HAART highly active treatment [**3**].

 The incidence of retinal microangiopathy in patients with advanced HIV disease is related to the severity of the immunodeficiency and is a bad prognostic sign [**5**].

 Less common but important causes of binocular vision loss in HIV / AIDS are varicella zoster virus retinitis and herpes simplex virus retinitis, ocular syphilis, ocular tuberculosis, ocular toxoplasmosis and ocular pneumocystosis [**6**].

 So far, there is no known study showing posterior segment ocular disease for HIV / AIDS patients in Romania. Thus, this study presents the posterior segment ocular manifestations of HIV / AIDS patients.

## Methods

There were 131 patients with HIV/AIDS included in the study, who presented to the Ophthalmology Office of "Matei Bals" National Institute of Infectious Diseases, Bucharest, from August 1, 2007 until August 1, 2012 with posterior segment ocular disorders. No case has been taken from other authors or other hospitals of the same profile.

 The study was conducted retrospectively, records of all the patients presenting with HIV / AIDS, who experienced posterior segment ocular disease were used for data analysis. The examination sheet of each patient registered the age, sex, number of CD4 copies/μL at presentation, adherence to treatment. A detailed ocular examination displayed visual acuity measurement, examination of the anterior segment and posterior segment of the eye. After sampling the biological probes (blood, conjunctival discharge on sterile environment), the etiologic agent of posterior segment ocular manifestations could be established.

 Visual acuity was recorded in Snellen eye chart through Snellen fractions. The examination of the ocular posterior segment included the examination of the vitreous and retina after the administration of mydriatics - tropicamide 1% by using the 90D lens for the slit lamp microscope and the 20D lens for the indirect microscope for a better visualization of the retinal periphery. Documentation of relevant changes at the vitreous and retinal examination was performed by fundus photography and fundus appearance diagrams.

 These individual data were analyzed and results were reported in percentage or absolute numerical value.

## Results

There were a high number of children and young people with HIV, who had posterior segment ocular disease. Most patients who have disorders of the posterior segment of the eye are in the age group 26-30 years (43 patients - 32.83%), followed by age group 19-25 years (31 patients - 23.66 %) and 15-18 years (27 patients - 20.61 %) (**Table 1**).

**Table 1:** The patients’ distribution according to age groups

Age groups	Number of patients
0-14 years	13
15-18 years	27
19-25 years	31
26-30 years	43
31-40 years	9
41-50 years	6
51-60 years	2

 Posterior segment ocular pathology is more common in AIDS patients (105 patients, 80.15%) than in HIV patients, which is justified by the reduced immune competence of the former and predisposition to diseases of infectious nature especially (**Table 2**). 

**Table 2:** Distribution of patients on antiretroviral therapy at ophthalmologic presentation

Known diagnosis at presentation	Number of patients with discontinued therapy	Number of patients under treatment
HIV	16	19
SIDA	10	85

 26.71% of the patients discontinued HAART therapy without the permission or without informing the infectious disease doctor. There is a greater compliance of AIDS stage patients regarding antiretroviral treatment (**Table 2**). 

 98.59% of the patients with CD4 less than 50 copies/μL are at AIDS stage versus 15.62% of the patients with CD4 counts between 100 and 199//μL who are also at AIDS stage (**Table 3**). 

**Table 3:** The distribution of the patients in the group according to the number of copies CD4/μL

Lymphocytes Count CD4/μL	HIV	AIDS
199-100	27	5
99-50	7	21
<50	1	70

 The representation according to the clinical diagnosis of the ocular posterior segment disorders is shown in Table 4. 

**Table 4:** The distribution of patients depending on the specific conditions of the posterior segment of the eye

Specific posterior segment ocular disease	Number of patients
Retinal microangiopathy	31
Retinitis	59
Choroiditis	8
Chorioretinitis	40

 A large number of patients (93) had ocular disease of retinitis and chorioretinitis (70.99%). Retinal microangiopathy was present in 30 patients (22.90%) (**Table 4**). 

 5 patients diagnosed with retinal microangiopathy had poor visual acuity at presentation, the rest (25 patients) showed no symptoms. In two patients, the soft retinal exudates characteristic for HIV retinal microangiopathy were accompanied by central retinal vein occlusion in one case and a main ram retinal vein occlusion in the other case. 

 The pathogen agents in cases of choroiditis were Cryptococcus in a case and Pneumocystis carinii in the other 7 cases, concomitant with respiratory impairment in patients who had neglected his treatment. It was noticed in all these cases that the absence of vitreous or iridociliary inflammatory reactions were explained in the context of total lack of defense capacity (average CD4 count < 25/μL). 

 Chorioretinitis were more numerous (40 cases), all of infectious cause, the pathogens being listed in Table 5. 

**Table 5:** Distribution of patients with chorioretinitis according to the etiological agent involved

Etiological agent	Number of patients
Treponema pallidum	6
Candida	7
Fumigatus	3
Oncocercus volvulus	3
Toxoplasma gondii	16
Mycobacterium tuberculosis	5

 The most numerous were toxoplasmosis retinitis by reactivation of preexisting latent infection due to the collapse of the defense capacity (CD4 < 50/μL in 12 patients and < 4/μL in 4 patients), especially since only 3 of the 16 were following the HAART treatment. 

 Luetic chorioretinitis were found in patients with secondary syphilis (4 patients) and tertiary syphilis (2 patients), undiagnosed until referral to an ophthalmologist, their evolution being favorable after starting treatment with penicillin. 2 patients had affected optic nerve and responded to treatment more slowly and partially. It was necessary to appeal to vitreoretinal surgery in patients with tertiary syphilis. 

 Fungal chorioretinitis involved Candida and fumigatus [**3**], in patients with CD4 < 200/μL and concomitant injuries of anterior ocular segment (iridocyclitis). 

 5 patients had tuberculous chorioretinitis evolving insidiously, only 2 presenting with follicular forms at the fundus examination. The clinical evolution of 4 out of 5 patients was retinal detachment. 

 The retinitis were numerous, the vast majority being viral (2 with varicella zostervirus and 48 with cytomegalovirus). Other 2 were bacterial, endogenously inoculated and one with Toxoplasma gondii. Their pathogenesis, except for the bacterial ones, was the reactivation of latent infections. 

 Varicella-zoster virus retinitis have occurred at a number of CD4 around 50/μL. Both cases responded poorly to specific treatment, one patient remained with a low visual acuity and the other evolved to retinal detachment, which recurred after surgical intervention. 

 CMV retinitis were diagnosed in patients with CD4 under 50/μL (one case with 199/μL). The clinical form thereof is shown in Table 6. 

**Table 6:** Distribution of patients with CMV retinitis depending on the clinical form shown

Clinical form	Number of eyes
Edematous form	29
Indolent form	5
Perivascular form	10
Optic neuropathy	4

 Evolution has often been poor under treatment, almost a third were unresponsive to sustained therapeutic measures. Thus, patients with optic neuropathy remained with very low or absent visual acuity, and 28% of the patients evolved to retinal detachments and secondary optic neuropathies. 

## Discussions

 We can sustain a specific immunological feature of patients studied, patients in the special condition of incapacity defense to infection, sometimes extreme (77.09% of the patients had a CD4 count below 100/μL). This condition explains clinical diversity and severity of eye disease. 

 Given the complexity of possible lesions in these patients with HIV / AIDS, especially those in advanced stages of the disease, ophthalmologic examination should be performed carefully, including indirect ophthalmoscopy through the dilated pupil entirely view of retinal periphery. 

 It appears that CMV retinitis were not associated with impairment of the anterior segment also underlining the importance of the absence of inflammatory reaction. In this respect, we believe that any patient with a CD4 count below 50 copies/μL should report to an ophthalmologist at every 3 months. 

 The simultaneous involvement of the anterior and posterior segment frequently signified a poor prognosis in prospect of visual acuity recovery, this being proven in 23 of the patients studied (fungal, toxoplasmosis and herpes infections). 

 Blindness affects 20.83% of the patients with CMV retinitis. The detection in time of ocular manifestations in HIV / AIDS patients can prevent severe loss of vision or can minimize it.

## Conclusions

It can be stated that HIV-positive patients may experience various symptoms that may further disabilitate them, so ophthalmologists should pay more attention to the HIV / AIDS patients and their ocular manifestations. Further studies are necessary to the understanding of risk factors or possible human genes involved in ocular complications arising from HIV / AIDS patients.
